# Autoselective transport of mammalian cells with a chemotactic droplet

**DOI:** 10.1038/s41598-020-62325-z

**Published:** 2020-03-26

**Authors:** Silvia Holler, Martin M. Hanczyc

**Affiliations:** 1 Laboratory for Artificial Biology, Department of Cellular, Computational and Integrative Biology (CIBIO), University of Trento, 38123 Trento, Italy; 2 Chemical and Biological Engineering, University of New Mexico, MSC01 1120, Albuquerque, NM 87131-0001 USA

**Keywords:** Biophysical chemistry, Biologics, Biomaterials, Chemistry, Fluid dynamics

## Abstract

Liquid chemical droplets, as models of artificial life, when pushed away from equilibrium possess some life-like behaviors such as fission, fusion, movement and chemotaxis. Chemotaxis, directed motion in response to external gradients, is typically an important process in living systems, but certain artificial systems are also capable of this activity. Previously it was shown that droplet-based chemotactic systems when interfaced with biological systems can act as transporters to move cargo such as hydrogel alginate capsules containing living cells. Here the effectiveness of our system to transport different mammalian cell lines (H460, H1299, A549, HEK293T and HS68) was tested. It was discovered that some lung cancer cell lines release surfactants only when placed in the hydrogel capsules. These surfactants establish the interface between the encapsulated cells and the droplet and also support the chemotaxis of the droplet. Because of this, the droplet-mediated transport system is selective for living cells that produce biosurfactants. This is an example of how the integration of artificial life and biological life could be designed where the systems augment each other and function together as a unit. In this case the living system produces the surfactants that the droplet needs for cargo transport and the artificial system provides the transport for the otherwise sessile mammalian cells. Future applications of droplet-based cell handling that is able to distinguish between cells based not only on viability but cell type, developmental stage or other quantifiable traits are considered.

## Introduction

Cells and organisms have evolved to respond to different types of signals: pressure, light^1^, temperature, pH^2^, oxygen^3,4^ and chemicals^5–7^. A well-described system of eukaryotic chemical sensing is the migration of *Dictyostelium amoebae* along an increasing concentration of cyclic adenosine-3${}^{{\prime} }$,5${}^{{\prime} }$-monophosphate (cAMP)^8,9^. If placed in steep cAMP gradients (more than 10${}^{-3}$ nM/$\mu$m), *Dictyostelium* is able to detect the spatial gradient and move towards the cAMP source^10^. Chemotaxis is very important as well in physiological processes such as organ development and maintenance^11^, embryogenesis^12^ and during the recruitment of inflammatory cells to sites of infection^13^ and inflammation^14^.

In fluid dynamics-based technologies, external flow fields have been shown to be useful for cell separation, cell interrogation and sorting. These technologies range from flow cytometry^15^ to electrode-mediated separation^16^. In these separation techniques, cells do not move in response to a stimulus-receptor-signal cascade mechanism but are controlled by the external environment in which they are situated. If cells are interrogated by fluorescence signal or incident light scattering intensity, they can be physically sorted within the flows based on a quantifiable cellular property^17^. These techniques require large superstructures that completely dwarf the fluids under scrutiny. Scaling down such devices has limitations if the actuation is effected by the instrument and not the liquids themselves.

It is therefore of interest to explore the dynamics and responsiveness of liquids themselves to understand potential technical applications. Droplets with fluid dynamical properties promote self-motion in response to chemical gradients. In particular, when a droplet of oil is added to an aqueous phase containing a surfactant, the surfactant quickly assembles a monolayer between the two immiscible phases. Certain chemical signals in the environment change the surface tension around the droplet due to the presence of the surfactant. For example it was found that fatty acids at the interface are sensitive to pH change^18^ and salt concentration^19^. When the droplet is exposed to external gradients, the imbalance of interfacial tension around the droplet results in fluid motion and convective flow due to a Marangoni instability. This allows the droplet to move directionally in the external chemical gradient. The chemotaxis is therefore dependent upon the presence of surfactant. In addition, the sensing, due to the surfactant at the interface, and actuation, due to the convective flow, are integrated in the individual droplets and not in any superstructure or instrument. It was previously demonstrated that this droplet system can be used to transport standard laboratory strains of cells such as *Escherichia coli* and *Bacillus subtilis*^20^. We wanted to expand this system to include the transport of mammalian cells lines. The transport of several proliferative cell lines was tested in our system: H460, H1299, A549, HEK293T and HS68. Many of the cell lines survived the transport and some survived only if a multilayered hydrogel was used. In the development of this system it was discovered that some cell lines produced biosurfactants during the application of the protocol that aided in the association of the encapsulating hydrogel with the motile droplet. As a consequence, this hybrid droplet-based transport system can successfully distinguish between living versus dead cells with selectivity emerging through the interface of artificial life systems with natural living systems.

## Results

### Transport of eukaryotic cells

**Figure 1:**
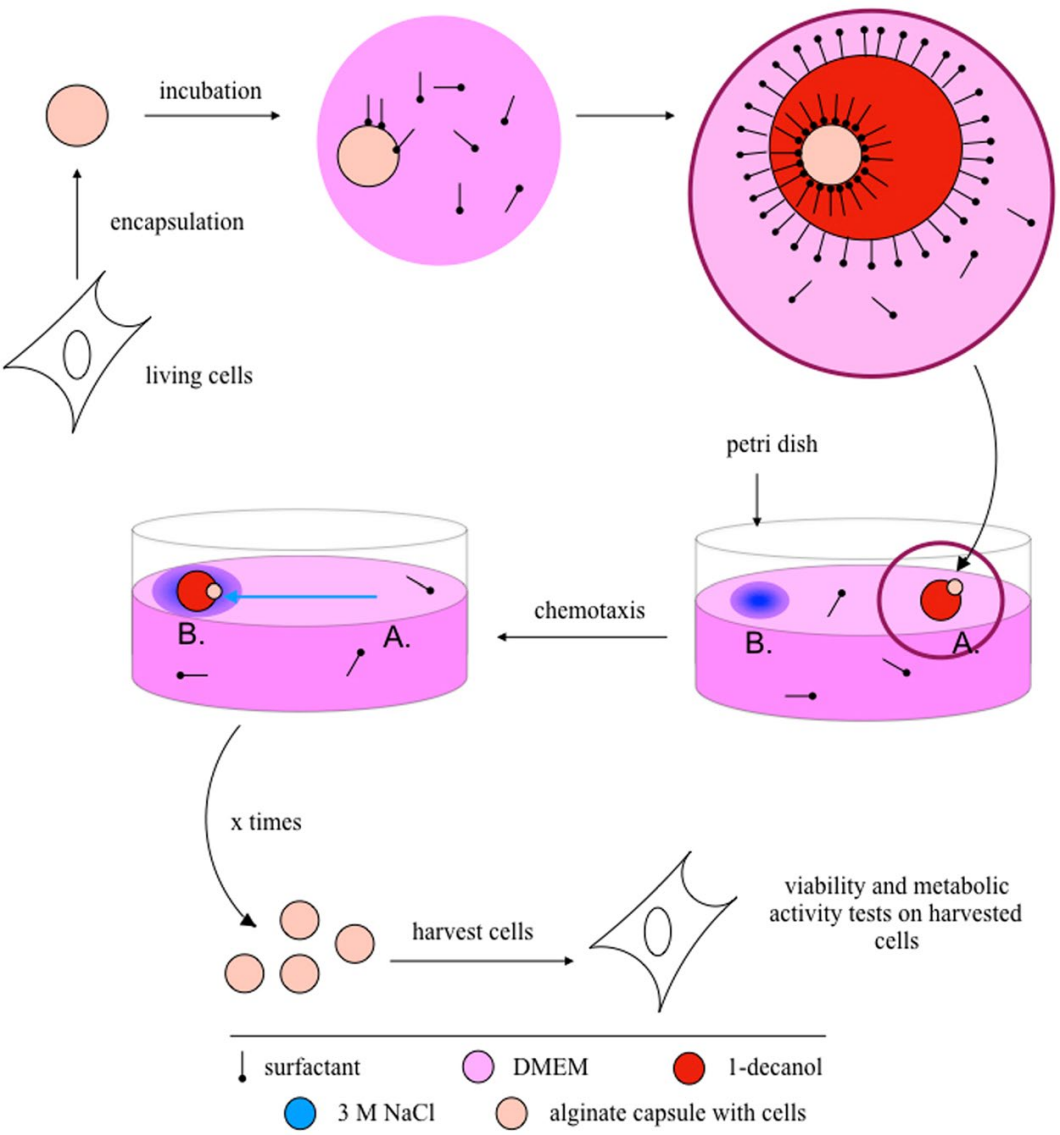
Autoselective capsule transport system. Cells were encapsulated in alginate capsules, incubated for two days in DMEM pure and then transported using a chemotaxic droplet. Both the transport and the adhesion of the aglinate capsule to the droplet depend on the production or supply of surfactant in the system. Afterwards several trials, cells were harvested and their viability and metabolic activity analyzed.

A transport system was employed to test whether cells placed in alginate capsules can be moved by chemotactic droplets and if they survive the process (Fig. 1). In the first step, cultured cells were pelleted and then encapsulated in alginate capsules (see Methods). The capsules containing eukaryotic cells have a diameter that varies between 2.5 and 3 mm. Their mean weight is 15.7 $\pm$ 0.6 mg (values obtained from 10 replicates of 10 capsules) with a volume of $\sim$15 $\mu$l. (A typical capsule is shown in Holler *et al*.^20^ Fig. S2). Individual capsules were manually positioned on individual 1-decanol droplets with red colorant floating on various aqueous phases, such as DMEM growth media or water supplemented with decanoate^20^. Then a chemical gradient was introduced by the addition of 3 M NaCl at a position distant from the droplet containing the capsule. The system was then monitored for droplet chemotaxis and transport. For an example of a chemotactic transport experiment, see Supplemental Movie S1. Finally the capsules were manually harvested from the experiment, hydrogel dissolved and the recovered cells tested for viability and metabolic activity.

Since hydrogels are hydrophilic materials, a key step in this transport system was the modification of the hydrogel to make it more hydrophobic and thus adhere to the surface of the decanol droplet. Previously, a solution of decanoate was used for this modification showing that the degree of hydrophobicity is controlled by the concentration of decanoate added to the hydrogel^20^. Various conditions for hydrogel modification were tested, because the cells packaged in the hydrogel capsule need to stay physically associated with the decanol droplet during the entire transport phase. If the association is not stable enough, the capsule will either be released at the origin or prematurely while the droplet is moving.

**Figure 2:**
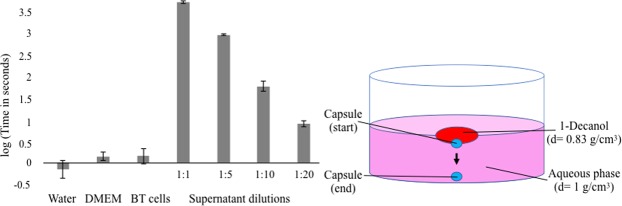
Bi-phase hydrogel association test. Capsules were prepared from alginate dissolved (5$\%$ w/v) in different pre-filtered aqueous phases. Capsules were manually placed upon a decanol droplet floating in DMEM aqueous phase and the times of the capsule-droplet association were recorded. For each condition 10 replicates were performed. Values for living cancer cells in capsules supernatant are the mean for the three lung cancer cell lines (A549, H1299 and H460) grouped together, as their individual values were nearly identical. Cells killed by bleach treatment (BT cells) were used as negative controls, and the reported value correspond to the mean for the three cells lines. Insert shows an explanatory diagram of the actual system (see Supplementary Figure S6).

**Figure 3:**
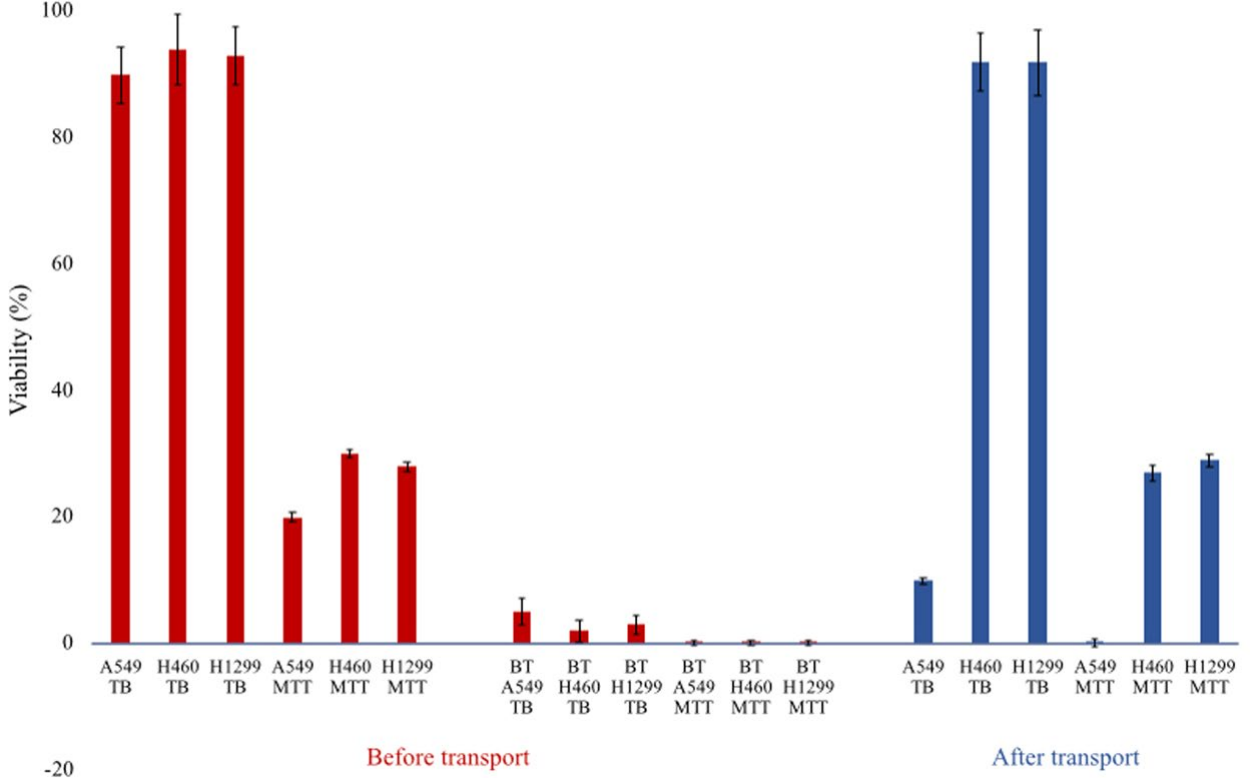
Viability and metabolic activity before and after transport. Viability results for Trypan Blue (TB) and 3-(4,5-Dimethylthiazol-2-yl)-2,5-Diphenyltetrazolium Bromide (MTT) tests on cells obtained from alginate capsule dissolution before and after transport. Bleach-treated (BT) were assayed for comparison. Error bars from experimental triplicates.

An easy assay was devised to quantify the association between 1-decanol and the hydrogel capsule by preparing a biphasic system consisting of the droplet transport system components (see Fig. 2). DMEM growth media as the aqueous phase was placed in wells of a 20-well plate. Then a 1-decanol oil droplet (density = 0.83 g/cm^3^) was placed on top of each aqueous phase. Capsules with a density greater than 1 were prepared under varying conditions (see below and Methods) and placed at the oil-water interface of this biphasic system. The amount of time that the capsule remained at the interface was recorded. The results of this assessment were in good agreement with the propensity of a capsule to be transported or lost during droplet-mediated transport. In addition several replicates for each condition (typically 10) can be quickly screened. Three different cells lines (A549, H1299 and H460) produced a supernatant in growth media that significantly affects capsule-droplet association. These three cell lines were first incubated in hydrogel capsules in DMEM for two days and this DMEM-based supernatant was then used in the production of the hydrogel capsules. After cross-linking, the capsules produced from different dilutions of this supernatant were manually placed in the biphasic test and the association times recorded. As controls, the association of alginate capsules produced in pure water, pure DMEM or supernatant from a 2-day incubation of capsules with bleach-treated cells (BT A549, H1299 or H460) were tested. Bleach-treated cells were produced by exposure to bleach (30 minutes) and then confirmed to be no longer viable (see Methods and results in Fig. 3). A summary of the time of capsule-interface association can be found in Fig. 2. The association times of the capsules produced from the supernatant that contained living cells (A549, H1299 and H460) with the decanol droplet were around 1 hour and 30 minutes, and the association time decreased with increasing dilution in water. This steady decrease of association time with dilution into water is consistent with the production and dilution of compounds responsible for the capsule-droplet association. The controls showed essentially no association of the capsule with the decanol droplet if either no living cells were present (growth media alone was not sufficient) or if the cells were killed by bleach treatment. Therefore, the results reveal that living cells encapsulated in hydrogel produced a supernatant containing compounds that highly increased the capsule-droplet affinity, and this can be used to tune the association time of the capsules with the droplets in the transport experiment.

In our previous study, decanoate was used as a surfactant to both modulate the hydrophobicity of the alginate hydrogel and as a chemotactic responsive molecule in the aqueous phase. To determine if the surfactants produced from lung cancer cells (as noted in the bi-phase hydrogel association assay) could substitute for all the decanoate in the system, the supernatant remaining from the incubation of capsules containing live lung cancer cells (A549, H1299 and H460) was tested. Supernatant obtained from two-day-old growth media containing encapsulated cells was placed in a 9 cm diameter Petri dish. A decanol droplet (of 20 $\mu$l) was added. A salt gradient was then introduced by adding 3 M NaCl to the aqueous phase^20^. For controls, pure DMEM and DMEM in which empty alginate capsules (no cells) or capsules containing dead bleach-treated cells were incubated for two days were tested. The supernatant in which capsules with living cells were incubated showed chemotaxis in all 10 out of 10 tested experiments. The controls did not show complete chemotaxis but only small or slow movements of the decanol droplets (see Supplementary Movie S2).

### Autoselective transport

Alginate capsules containing living cells can produce their own surfactants that allow the hydrogel to adhere to the decanol droplet (Fig. 2) and that support chemotaxis. Can the biosurfactants from the lung cancer cells can be used for both hydrogel capsule adhesion and chemotactic transport of an alginate capsule in the same experiment? Alginate hydrogel was polymerized using DMEM supernatant (mixed 1:1 with water) from incubated and encapsulated lung cancer cells as described above, and the capsules were placed on the decanol droplets for transport. Capsules polymerized using lung cancer cell-DMEM supernatant can be efficiently transported by decanol droplets in every trial (10 successful transports out of 10 transport trials).

The natural surfactants produced by the cell lines can modify the hydrogel surface allowing for adhesion to the decanol droplet and the surfactants can support droplet chemotaxis. Perhaps the system could distinguish between living and dead cells. This is important as the surfactants that modified the hydrogel could have been produced from the living cells or from the remains of dead cells. To kill the cells, the cells were treated with bleach. Viability measurements after treatment confirmed that the cells were no longer viable, Fig. 3. Then the transport of capsules was performed with living lung cancer cells (A549, H460 and H1299), bleach-treated (BT) lung cancer cells and empty capsules (no cells). Only the capsules with viable cells can be transported using decanol droplets (10 effective transports out of 10 trials for each cell line, see Supplementary Movie S1). Empty capsules and capsules with BT cells are released from the droplet as soon as they are placed upon it (10 releases out of 10 trials, 10 trials for each cell line). Therefore the droplet transport system is not only selective for cells lines that produce surfactants but is autoselective for the transport of living lung cancer cells.

### Cell survival after transport

Cells in alginate capsules can produce surfactants that govern the association of the capsule with the decanol droplet and that act as a chemo-sensitive molecules that support droplet chemotaxis. Therefore it is sufficient to encapsulate living cells in alginate capsules, incubate the capsules with cells in growth media, and then use the capsules and the supernatant to perform chemotaxis experiments successfully. However in our previous paper it was reported that not all cells survive the transportation step^20^. Therefore the mammalian cells lines once transported were then assayed for viability, metabolic activity and proliferation.

Lung cancer cells (A549, H1299 and H460) were encapsulated in alginate and transported in the droplet chemotaxis system, Fig. 1. Capsules with roughly 10 * 7 cells each, were transported, harvested and analyzed. In the case of capsule release and decoupling from the droplet, 0.2 M decanoate at pH 12 was added manually near the droplet^20^. Capsules were harvested up using tweezers, dissolved using sodium isocitrate and cell viability and metabolic activity before and after the transport were tested using Trypan Blue (TB) staining and 3-(4,5-Dimethylthiazol-2-yl)-2,5-Diphenyltetrazolium Bromide (MTT) test. Using Trypan Blue count, cells were plated in a specific cell density and their proliferation microscopically assessed. The results of viability and metabolic activity using TB staining and MTT tests after capsule dissolution before and after the transport are reported in Fig. 3. Lung cancer cells killed by bleach treatment (BT) were assessed for comparison.

The cell lines H460 and H1299 showed high viability levels with TB but lower metabolical activity using MTT after the transport. This is likely due to the two days of incubation of the cells in alginate gel. The cell membranes were still intact but the metabolic activity was lowered. A549 cells instead showed low viability and low metabolic activity levels after transport. This is likely due to the high toxicity of decanol for this cell line^21^. In addition, A549 cells when incubated under very favorable conditions on Petri dishes ($3{7}^{\circ }$C and 5$\%$ CO${}_{2}$ with DMEM complete after capsules dissolution in sodium isocitrate) no longer proliferated after transport. Instead, H460 and H1299 proliferated efficiently before and after the transport which demonstrates that different cell types have varying sensitivity to this technique. The cells retrieved from capsules before transport reached confluence (plated 10 * 6 cells in each well, in 6 well plate) in two weeks. The cells retrieved from capsules after transport reached confluence in three weeks under the same plating conditions. Metabolic activity was checked with MTT after two and three weeks respectively and the mean viability was 88$\%$ (cells in capsules plated before transport: H460 92 $\pm$ 3$\%$ and H1299 85 $\pm$ 3$\%$; and after transport: H460 91 $\pm$ 3$\%$ and H1299 83 $\pm$ 2$\%$). Mean and standard deviation were obtained from experimental triplicates. All controls with bleach treated cells showed almost null viability values (max 5$\%$) with TB and MTT. When the 0.2 M decanoate decoupler was added to the system, this decreased the mean viability of the cells by 15–20$\%$ (assessed by trypan blue staining and MTT test). Since A549 cells showed low viability and low metabolic activity levels after transport, the viability of A549 cells within an alginate capsule itself was assessed. Calcein AM and propidium iodide staining was used and visualized using confocal microscopy. Capsules before and after transport, including capsules sectioned to allow for more complete staining, showed high cell viability (visualizable by the green color of calcein AM in Supplementary Fig. S1). It is possible that the cells inside the hydrogel capsules were blocked in G0 phase as they appeared roundish, and this would explain why A549 cells while still viable were not able to proliferate properly after transport.

### Surface tension, transport efficiency and speed

**Figure 4:**
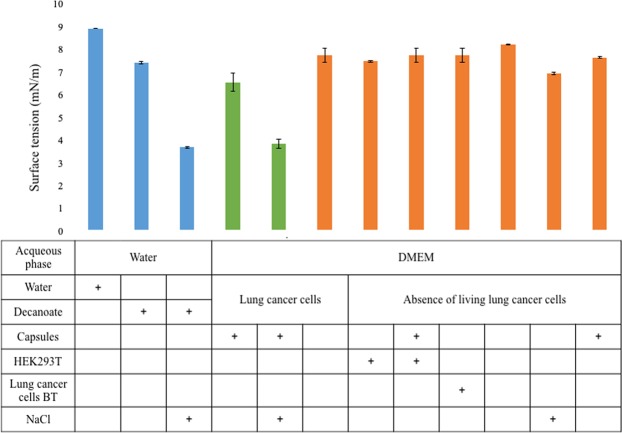
Surface tension of 1-decanol in different aqueous phases. Surface tension (mN/m) for 1-decanol was determined by the inverted pendant drop method in: water, decanoate 5 mM pH 11.5, decanoate 5 mM pH 11.5 mixed 1:1 with 3 M NaCl, DMEM with 10 * 7 lung cancer cells (A549, H1299 and H460) in capsules, this same supernatant mixed 1:1 with 3 M NaCl, DMEM with 10 * 7 lung cancer cells (A549, H1299 and H460 without capsules), DMEM with 10 * 7 HEK293T cells, DMEM with 10 * 7 HEK293T cells in capsules, DMEM with 10 * 7 lung cancer cells bleach treated (lung cancer cells BT: A549, H1299 and H460), DMEM, DMEM mixed 1:1 with 3 M NaCl and DMEM incubated with empty alginate capsules. All the DMEM aqueous phases were incubated for 2 days at $3{7}^{\circ }$C with or without cells or capsules. Decanol droplet volume was typically 10–11 $\mu$l. The blue bars represent for our previous transport system using decanoate as the salt-sensitive surfactant^20^. The green bars represent the best performance of chemotaxis found in this study. The bars in orange are controls for reference. Error bars correspond to standard deviation of 10 droplets for conditions without cells, 10 droplets with three experimental replicates for HEK293T, for each lung cancer cell type and for each lung cancer cell type bleach treated (the mean from the three lung cancer cell lines is reported). Specific values for each lung cancer cell line are plotted in Fig. 5 and Supplementary Fig. S3.

**Figure 5:**
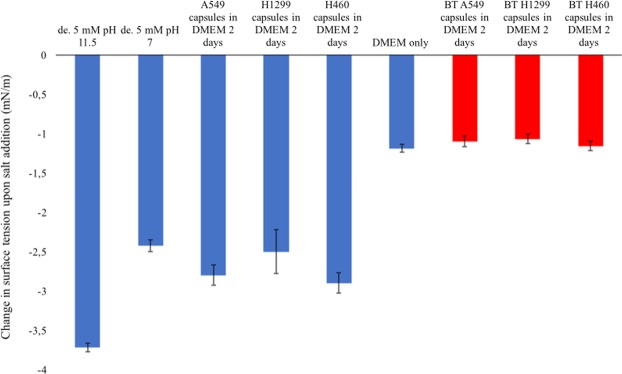
Change in surface tension of 1-decanol in different aqueous phases after the addition of salt. 3 M NaCl was mixed with the various aqueous phases in 1:1 ratio, see Methods. All the supernatants were obtained from two-day incubations of 10 * 7 encapsulated cells. Error bars correspond to standard deviation on 10 replicates of each experimental replica (3 for each cell line in alive or bleach-treated (BT) condition). Negative controls with bleach treated lung cancer cells are reported in red.

Chemotactic decanol droplets move towards regions of higher salt concentration, and this is mitigated through differences in surface tension when NaCl is added to the system. Several different aqueous phases were assessed for this difference by pendant drop tensiometry with pure 1-decanol. Surface tension values, for these aqueous phases with and without salt addition and controls, are reported in Figs. 4, 5 and Supplementary Fig. S3. For terminology, DMEM or DMEM pure refers to the DMEM freshly prepared and sterilized, supernatant refers to the DMEM pure in which the capsules with mammalian cells have been incubated for two days, and DMEM complete refers to DMEM pure with the addition of Pen-Strep (1$\%$), L-Glutamine (1$\%$) and FBS (10$\%$).

Figure 4 shows the surface tension values (mN/m) for decanol placed in the different aqueous phases. The highest surface tension value is for pure decanol in pure water. Our previous experiments with motile decanol droplets were performed with decanoate as the chemo-responsive surfactant^20^, and surface tension values of decanol in decanoate with and without salt addition are shown as the blue bars. In the current study, the surface tension of decanol in various aqueous phases that potentially contain biosurfactants was assessed. The most obvious (and statistically different) change in surface tension was from the supernatant where hydrogel-encapsulated lung cancer cells were incubated (green bars, Fig. 4; the associated probabilities for Student’s t-test were the following: 1.55974E-60 for DMEM vs surfactant of A549 capsules, 6.12539E-47 for DMEM vs H1299 capsules and 8.49829E-63 for DMEM vs H460 capsules). When lung cancer cells were not encapsulated, HEK293T cells were encapsulated instead, or dead bleach-treated cells were used, little or no variation in the surface tension was detected. This confirms the presence of tensio-active compounds produced by living lung cancer cells only when they are encapsulated in the hydrogel.

Next the decrease in surface tension after salt addition to these various aqueous phases was assessed (Fig. 5). The assays included our previously employed decanoate system at alkaline pH. Then the pH was decreased to 7 to make the system more compatible with growth conditions for mammalian cell lines. With decanoate at pH 7 as the aqueous phase, chemotaxis was confirmed by performing droplet motion tests in salt gradients as described above. Then the surface tension variation for decanoate pH 11.5, pH 7 and for all the lung cancer cell supernatants was characterized. Various aqueous phases were mixed with 3 M NaCl 1:1 to reproduce a situation similar to the salt addition point in a chemotaxis experiment. In the case of decanoate 5 mM pH 11.5^20^ the surface tension decrease after mixing with 3 M NaCl (1:1 ratio) is 3.7 mN/m. For decanoate pH 7 the decrease is 2.5 mN/m. When the supernatant from capsules with live cells was mixed with NaCl the surface tension decrease is 2.8 mN/m. When the supernatant from capsules with dead bleach-treated cells was mixed with NaCl the value was 1.1 mN/m (Fig. 5, red bars). Therefore the most dynamic response in the change in surface tension was found with the aqueous phase containing decanoate at pH 11.5 followed the supernatant from encapsulated lung cancers cells (A549, H1299, H490). In contrast almost no surface tension change was found with the supernatant from dead lung cancer cell lines.

The chemotactic perfomance of droplets in the various aqueous phases above was analyzed, and chemotactic performance (such as velocity) versus change in surface tension upon salt addition was plotted. It was found that a larger decrease in surface tension upon salt addition correlates with a better chemotactic performance (Supplementary Fig. S4). It is noted that even when using aqueous phases with a small decrease in surface tension upon salt addition (DMEM only or dead bleach-treated cells) limited chemotactic movement could still be possible. A DMEM only control was tested for droplet movement. Small fluctuations in droplet motion towards the salt source were noted but the chemotactic response (distance and velocity) was much reduced. An example of limited chemotaxis of 1-decanol in DMEM only can be found in Supplementary Movie S2 https://youtu.be/HSOKL08viXs. The motion and the mean path length of chemotaxis of decanol in DMEM only was the 56 $\pm$ 3$\%$ of the total distance between droplet and salt starting point and the mean velocity was 0.0131 cm/sec $\pm$ 0.002 (1/5 of the normal decanol in decanoate velocity^20^). In contrast, the decreases in surface tension for decanoate pH 7 and 11.5 and live cell supernatant were however greater after NaCl addition (Fig. 5 and Supplementary Fig. S3). This difference in surface tension upon salt addition correlated well with the good performance of droplet chemotaxis under these conditions (Supplementary Fig. S4).

### Double encapsulation

Some cell lines were more sensitive to the experimental conditions, as they were either not viable or did not proliferate after transport. To solve this problem, a double encapsulation system was devised: a shell of alginate dissolved in A549 supernatant (diluted 1:1 with water) was formed around the original capsule containing the cell cargo (see Supplementary Figure S5). Then transport experiments were performed as before. After transport each capsule was harvested with tweezers and its outer alginate shell manually removed. Capsules were then dissolved and cell viability assayed through TB staining and MTT test (Table 1). The standard error was generated from 3 experimental replicates of the transport of 10 * 7 cells in capsules. The control value to normalize the MTT was obtained from healthy cells assayed just before encapsulation and transport.

**Table 1:** Proliferation of HEK293T and HS68 cells after transport using single or double encapsulation.

Cell Line	Single encapsulation			Double encapsulation		
	Trypan Blue	MTT	Proliferation	Trypan Blue	MTT	Proliferation
**HEK293T**	22 $\pm$ 6%	3 $\pm$ 0.8%	no	89 $\pm$ 4%	87 $\pm$ 7 %	yes
**HS68**	6 $\pm$ 2%	2 $\pm$ 0.6%	no	51 $\pm$ 5%	56 $\pm$ 8 %	yes

As shown in Table 1, when both HEK293T and HS 68 cells were transported using the protocol reported above, they had very low viability and no longer proliferated. However, double encapsulation allowed not only transport but also protection for the encapsulated cells. In all the transport experiments, the double encapsulation showed affinity for the decanol droplet and the transport was efficient. The average survival for HEK293T cells was around 89$\%$ and for HS 68 was 51$\%$. This viability reduction for fibroblasts was shown even when these cells were incubated at room temperature for a time corresponding to the whole encapsulation and transport protocol. Both cell lines were still able to proliferate after the transport. MTT and TB viability assays on cells obtained after transport using only a single encapsulation showed that almost all transported cells were killed during the transport. Notably, the viability problem with these two cell lines was already apparent independent of the transport system as these two cell lines die after two days of incubation in DMEM. However using a double encapsulation protocol almost completely obviates this problem and allows for successful transport and delivery of viable cells.

## Discussion

Previously we demonstrated the successful transport of bacterial and yeast cells^20^. Now this technology was extended to integrate the transport of mammalian cell lines, as a proof of principle of motile droplet-mediated transport. During the course of developing the system, the natural tendency for some cells to produce their own surfactants was exploited. Not only do the surfactants produced naturally by these cell types eliminate the need to add exogenous surfactant to our system, but these cell line-derived surfactants function as both the chemotactic responsive element and the droplet-hydrogel mediator. The cells lines were successfully transported in every trial and showed adequate survivability. For those cell lines that did not survive the protocol, a simple double encapsulation with hydrogel allowed for transport, viability and proliferation.

This is a demonstration of how two very different systems once integrated can function together as a unit. In this way mammalian cell lines that were non-motile can move chemotactically once paired with the droplet. This ability may play future roles in tissue repair and reconstruction. Cell lines packaged in hydrogel capsules produce surfactants allowing for the entire system to operate. Therefore this technological system is able to distinguish between living lung cancer cells due to their response to specific external conditions. In this case alginate encapsulation promotes surfactant secretion which then supports the chemotaxic movement.

Without packaging the cells, the cells would die and no surfactant would be produced. In this way the chemotactic droplet system was able to selectively transport live cargo. This is a primitive example of how self-motile systems may be produced that can also be selective for the types of cargo transported. Perhaps in the future the selectivity could be extended to demarcation between different types of mammalian cells (in principle this system could already distinguish between mammalian and bacterial cell types) due to stage of differentiation, production of key markers, and perhaps potency. Since the sensing of the environment and the movement in the system are provided by the droplet itself, the directed and selective transport of cells occurs locally and at a small scale. No extensive apparatus such as a cell sorter or even microfluidics platforms with external pumps are necessary. This then would allow for remote applications where the inclusion of heavy infrastructure would not be desirable or even possible due to weight, energy requirements or maintenance demands. In addition the complete system becomes more autonomous as the activity of the droplet comes from self-generated fluid dynamics. This type actuation as a technology is more commensurate with how living systems move and integrate into larger scale structures and environments.

## Materials and Methods

### Materials

All reagents were supplied by Sigma Aldrich: decanoic acid, 1-decanol, Oil red O, sodium hydroxide, sodium chloride and sodium alginate. Glass DURAN Petri dishes were supplied by Fisher, syringes and needles from PIC and cell culture polystyrene dishes and 96 well plates from Corning. Dulbecco’s Modified Eagle’s Medium (DMEM), Phosphate Buffered Saline (PBS), Fetal Bovine Serum (FBS), L-Glutamine (L-Glu), Penicillin-Streptomycin solution (Pen-Strep) and Trypan blue dye were provided by Invitrogen and 3-(4,5-Dimethylthiazol-2-yl)-2,5-Diphenyltetrazolium Bromide (MTT) was provided by Vinci Biochem.

### Methods

Unless otherwise specified, experiments were carried out at room temperature. The water used in the experiments was *milliQ* purified.

### Alginate hydrogel and capsule formation

Alginate capsules for transport were prepared by first dissolving sodium alginate (5$\%$ w/v) in DMEM by magnetic stirring and for sterility reasons the obtained gel was kept in a heat bath for 2 hours at $7{0}^{\circ }$C. Cells to be transported were grown until confluence in DMEM complete (DMEM high glucose with the addition of 1$\%$ Pen-Strep, 1$\%$ L-Glutamine and 5$\%$ FBS), resuspended, counted and checked for viability. Cells and their viability were quantified using Trypan Blue dye, countess cell counting chamber slides by Life Technologies and countess automated cell counter by Invitrogen. Cells were then pelleted and resuspended in fresh DMEM pure to a final concentration of 2 $\times$ 10 * 7 cells per 1 ml (centrifugation: 1500 rpm, 5 min). 1 ml of resuspended cells was mixed with 5 ml of sodium alginate gel. The alginate mix was loaded into a syringe (needle 25 G $\times$ 5/8”, 0.50 $\times$ 16 mm). During this step almost half of the gel volume was lost. The alginate mix with cells was manually pushed out of the syringe, drop by drop, into 1$\%$ w/v CaCl${}_{2}$ for cross-linking. The capsules that formed were left to cross-link for five minutes, washed twice using DMEM and then incubated for two days in DMEM (in controlled conditions, $3{7}^{\circ }$ and 5$\%$ CO${}_{2}$). This procedure was also followed for capsules containing BT cells (pellet was resuspended 30 minutes in bleach to kill the cells) and for empty capsules (no cells added). For control empty capsules fresh sterile growth media was added to the alginate mixture instead of pelleted cells.

### Bi-phase hydrogel association test

The supernatant was retrieved from the incubation of alginate capsules in DMEM. It was filtered using 0.22 $\mu$m syringe filters in polyethersulfone (PES, by Euroclone) and mixed with water in different quantities: 1:1, 1:5, 1:10 and 1:20. Sodium alginate was dissolved in these solutions at 5$\%$ w/v. A homogeneous gel was obtained through magnetic stirring and afterwards cross-linked in 1$\%$ w/v CaCl${}_{2}$. The obtained capsules were dried for 10 minutes in a biological hood in preparation for the association test. Each well of a 20 well plate dish was filled with 1 ml of aqueous phase and 200 $\mu$l of 1-decanol was added on top of the aqueous phase. Capsules were manually placed, using tweezers, upon the decanol droplet and the time for capsules to fall to the bottom of the well plate was recorded.

### Transport and cell viability assessment

Petri dishes were filled with 9 ml of the supernatant: DMEM aqueous phase incubated with capsules with lung cancer cells for two days. 250 $\mu$l 1-decanol droplet colored with Oil Red O (1 mg/ml as a colorant) was placed on the supernatant. 10 alginate capsules (dried for 10 minutes in a biological hood) were manually placed upon 1-decanol droplet and then transported through the creation of a salt gradient (1 ml of 3 M NaCl). Capsules were then released by manual addition of decanoate (0.2 M pH 12) or picked up using tweezers. This procedure was repeated until all the capsules (120–130 capsules, 10 * 7 cells in total) were transported. Capsules were washed with DMEM, dissolved using 50 mM sodium isocitrate (shaking incubation) for 15 minutes, centrifuged, pelleted and resuspended in 100 $\mu$l PBS. Cell count and their viability were quantified using Trypan Blue dye, countess cell counting chamber slides by Life Technologies and countess automated cell counter by Invitrogen. MTT was performed to check for metabolic activity using a 96 well cell culture cluster Costar 3596 (by Corning Incorporated). 10 * 5 cells in PBS were placed in each well, 10 $\mu$l of MTT added and placed in a dark incubator ($3{7}^{\circ }$C and controlled Co${}_{2}$ for 3 hours). After 3 hours, solution was poured away form each well and 100 $\mu$l DMSO added. Reading using TECAN Plate reader (measurement wavelength 570 nm and reference background wavelength 690 mm) was performed with PBS used as negative control. For the final check for viability, cells obtained before or after the transport were plated in a 6-well cell culture cluster Costar 3516 (by Corning Incorporated) at 10 * 6 cells/well to check for their proliferation over time.

### Capsule staining and microscopy

Capsules were visualized by microscopy at two different stages: after 2 days of incubation in DMEM and after transport. Capsules were incubated in calcein AM and propidium iodide staining solution (1:1000 and 1:500) for 1 hour. Capsules were then visualized by confocal microscopy, Nikon A1 (Nikon Instruments, The Netherlands). For sectioning, the capsules were manually cut using a spatula.

### Chemotaxis feasibility and autoselectivity

For each aqueous phase 10 chemotaxis experiments were performed. DMEM and DMEM in which empty alginate capsules or capsules with dead cells were incubated for two days were used as controls. A Petri dish (90 mm diameter) was filled with 9 ml of aqueous phase and a 20 $\mu$l 1-decanol droplet colored with Oil Red O (1 mg/ml as a colorant) placed on the aqueous phase. Chemotaxis was assessed through the addition of 400 $\mu$l of 3 M NaCl to the system using a micropipette.

To test the complete transport system. Alginate hydrogel was polymerized with DMEM supernatant (mixed 1:1 with water) from incubated and encapsulated cells (A549, H1299 or H460, 10 * 7 cells). Each capsule was placed on the decanol droplets (20 $\mu$l) for transport and 400 $\mu$l of 3 M NaCl added to the system on the opposite side of the dish as the chemoattractant. The aqueous phase used for the transport was obtained from DMEM incubated with living cells encapsulated in hydrogel for two days (same aqueous phase used to polymerize alginate after mixing with water). Capsules used as negative controls were polymerized using the DMEM supernatant of capsules that did not contain cells, contained bleach-treated cells (A549, H1299 or H460), or cell lines that do not produce surfactants (e.g. HEK293T). For each condition ten transport replicates were performed. Using this system chemotaxis selectivity for different cargo was tested. To assess if the chemotactic droplet system was able to differentiate between different types of cargo, the transport of capsules with living lung cancer cells, empty capsules (no cells) and capsules with bleach-treated lung cancer cells (dead cells) was tested. Capsules were created using the previously described protocol. For empty capsules, fresh DMEM was added to the alginate solution. For BT cells, the cells were counted and adjusted to 10 * 7 cells per ml (same density as the living cells), pelleted, and then resuspended in bleach for 30 minutes. After 2 days of incubation in DMEM ($3{7}^{\circ }$C and controlled CO${}_{2}$), capsules were ready for transport. 10 transport experiments were performed for each capsule type, and their own supernatant was used as transport environment, as described above.

### Surface tension, transport efficiency and speed

Using Theta-lite tensiometer by OneAttension, Biolin Scientific (Nordtest Srl, Italy) the surface tension of an inverted pendant drop of 1-decanol (needle 22 G) in different aqueous phases was assayed. The change in surface tension upon chemoattractant salt addition was measured: 3 M NaCl was mixed with the various aqueous phases in 1:1 ratio. Error bars correspond to standard deviation of 10 replicates (10 decanol droplets) of each experimental replica. In the case of sample containing biological material such as cells three experimental replicates were performed (from each of them 10 droplet replicates were analyzed). Droplet motion was recorded using C920 Logitech HD Pro Webcam and distance and velocity values were obtained from the videos using a free software: ’Tracker - Video Analysis and Model Tool’ (see https://physlets.org/tracker/). The video analysis was performed by setting a reference measure from the ’real object’, in this case the 9.00 cm of the Petri dish diameter.

### Double encapsulation and cell transport

Doubly encapsulated cells were made in two steps. First, capsules were created: 4 ml of sodium alginate 2$\%$ w/v (in DMEM complete mixed 1:1 with water) was mixed with 1 ml of resuspended cells ($2\,\times \,10$ * 7) and cross-linked in CaCl${}_{2}$ 1$\%$ w/v. The CaCl${}_{2}$ solution was afterwards poured away, and capsules were picked up one by one using sterile tweezers and rolled on an alginate gel 5 $\%$ w/v (in A549 supernatant 1:5 mixed water). After coating, the capsules were added to a CaCl${}_{2}$ solution for a second cross-linking, creating a hydrogel shell surrounding the capsule. The effect of this double encapsulation was tested on two different cell lines: HEK293T and HS 68. Cells with double encapsulation were transported by a droplet in a Petri dish as described above. Viability (Trypan blue), metabolic activity (MTT), and proliferation were assayed before and after the transport using both single and double encapsulation. Three experimental triplicates were performed for each cell line and condition.

## Supplementary information

Supplementary Information.
