# Supplementary material for: Autoselective transport of mammalian cells with a chemotactic droplet
Source: Sci Rep. 2020 Mar 26;10:5525. doi: 10.1038/s41598-020-62325-z (PMC7099059; doi:10.1038/s41598-020-62325-z)
Supplement: Supplementary file 1 — Supplementary Information. [file 41598_2020_62325_MOESM1_ESM.pdf]

Autoselective transport of mammalian cells with a chemotactic droplet

Silvia Holler[1] **and Martin M. Hanczyc**[1,2,*]

[1]Laboratory for Artificial Biology, Department of Cellular, Computational and Integrative Biology (CIBIO), University of Trento, 38123, Trento, Italy
[2]Chemical and Biological Engineering, University of New Mexico, MSC01 1120, Albuquerque, NM 87131-0001, USA
[*]martin.hanczyc@unitn.it

ABSTRACT

Supplementary Movie S1

Alginate capsule transport with living cells, 30x real time. The chemotaxis experiment is performed in a glass Petri dish with 9 cm diameter. A 1-decanol droplet is placed in the experiment and an alginate capsule (of 2 mm diameter) containing living cells placed upon it. The droplet migrates towards the point of salt addition (400 μl 3 M NaCl) carrying the alginate capsule. The experiment is captured on video from above. https://youtu.be/DH2O2XuzFpQ

Supplementary Movie S2

Decanol droplet placed in pure DMEM and addition of 400 μl 3M NaCl, 10x real time. The experiment is captured on video from above. https://www.youtube.com/watch?v=HSOKL08viXs&list=UU--ke14e3t_HiuUfMowfsRg&index=2

Supplementary Figure S1

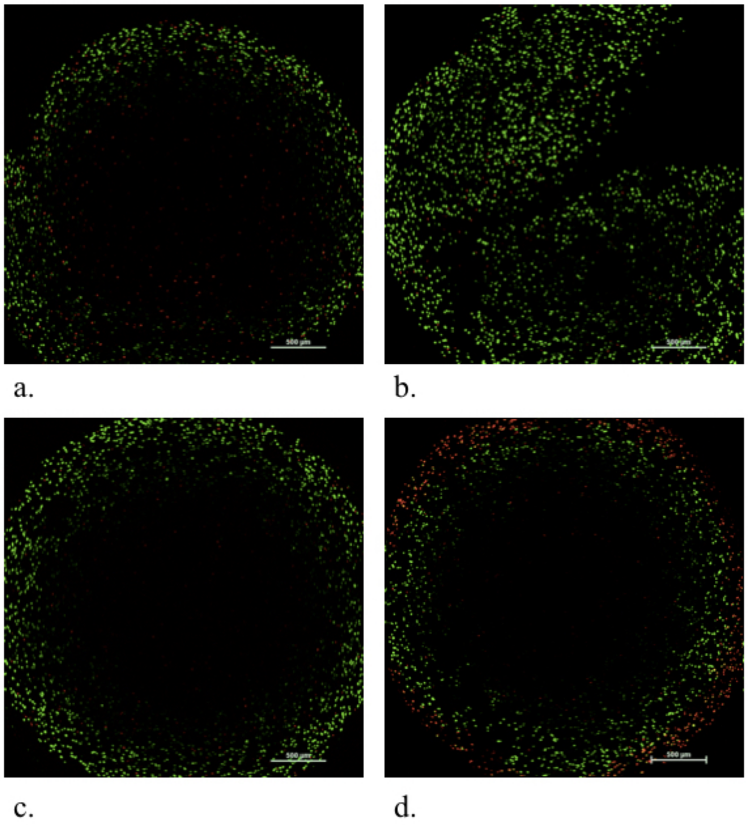

Staining for living and dead cells in alginate capsules. Calcein AM (green) and PI (red) staining of capsules pre-transport (a), sectioned capsule pre transport to allow the stain to penetrate (b), capsule post-transport (c), and capsule post-transport after using 0.2 M decanoate pH 12 to release the capsule cargo (d). Calcein AM stains living cells that will appear in the images as green and propidium iodide stains dead cells appearing red. The staining was performed on capsules containing A549 cells isolated before and after transport (in A549 capsule aqueous phase). Images were obtained using Nikon A1 (Nikon Instruments, The Netherlands).

Supplementary Figure S2

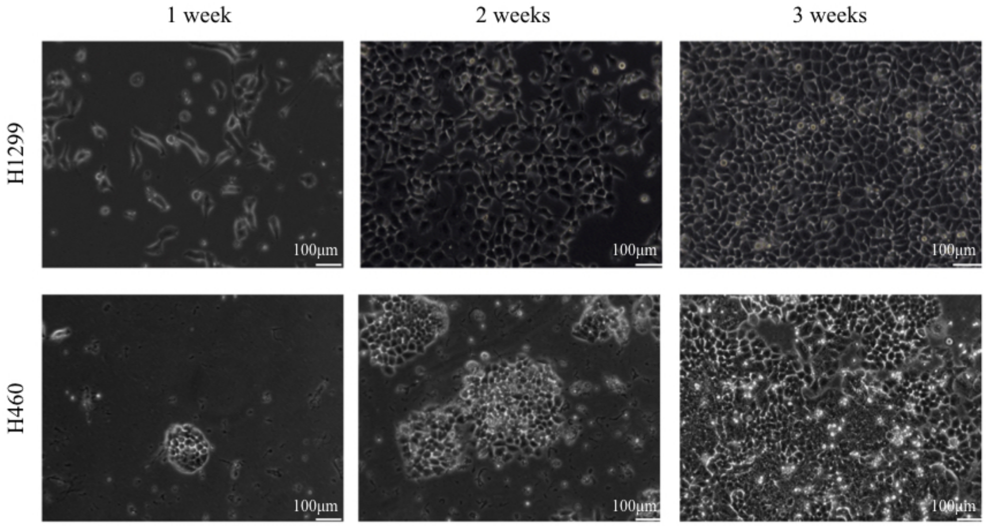

Optical image of H460 and H1299 plated after transport (after 1, 2 and 3 weeks respectively). Images were obtained using Leica DM IL LED Fluo microscope (Leica Microsystems CMS Gmbh Wetzlar).

Supplementary Figure S3

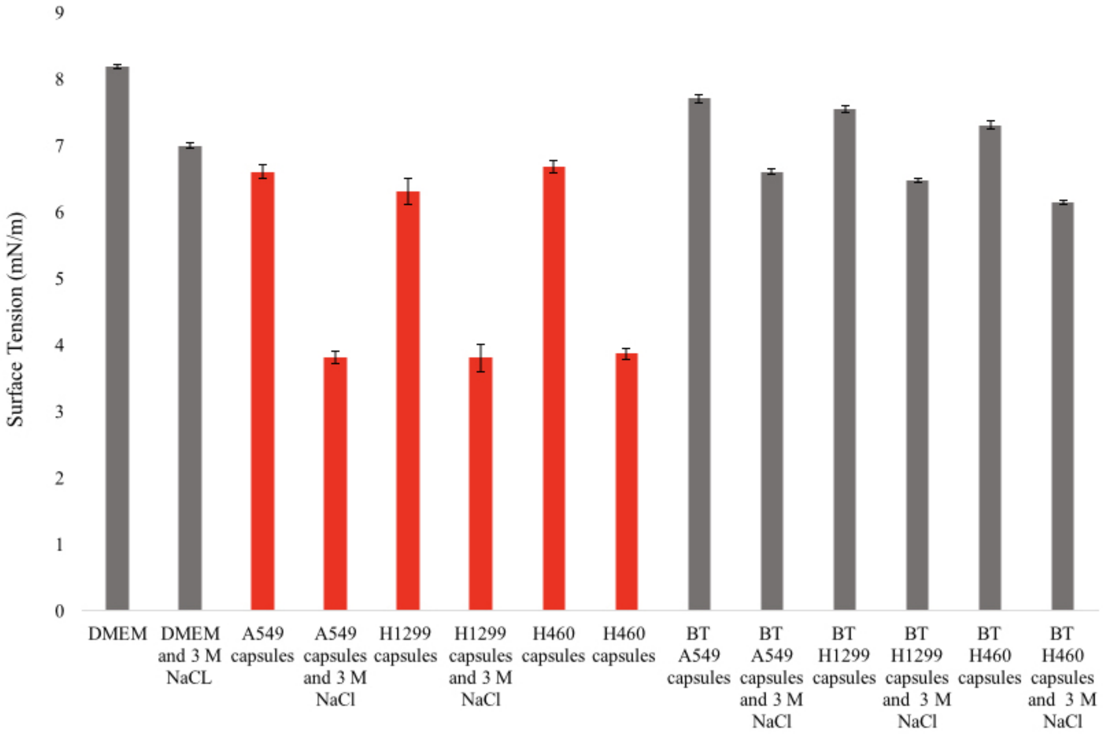

Surface tension of 1-decanol in different aqueous phases before and after the addition of salt. 3 M NaCl was mixed with the various aqueous phases in 1:1 ratio, see Methods. All the supernatants were obtained from two days incubation of 10*7 encapsulated cells (alive or bleach-treated (BT)). Error bars correspond to standard deviation on 10 replicates (10 drops) of each experimental replica (supernatant). Three experimental replica for each cell line in alive or bleach treated condition were analyzed. Negative controls with DMEM or bleach treated lung cancer cells are reported in grey.

Supplementary Figure S4

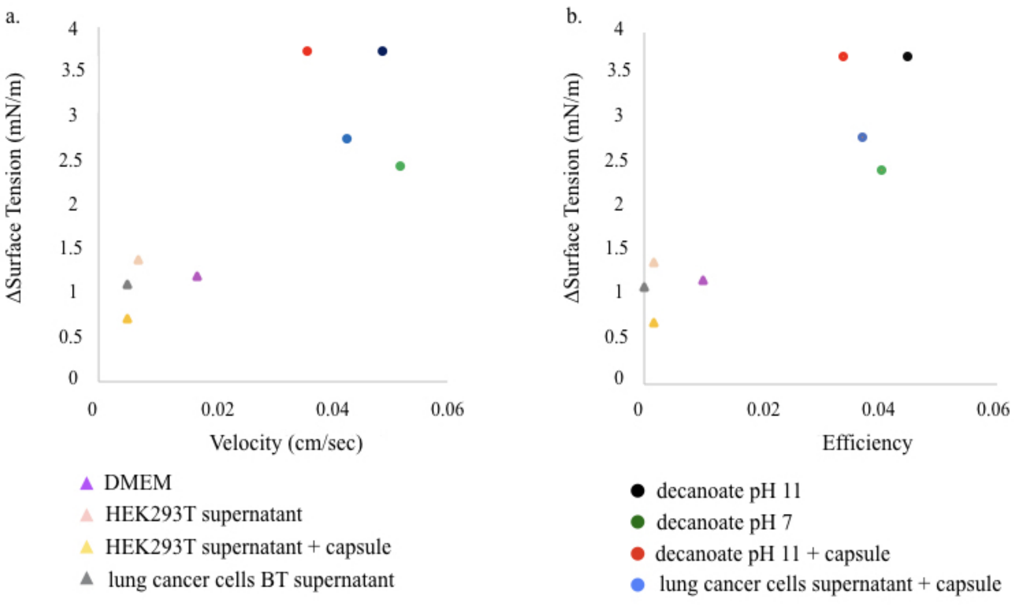

Chemotactic performance versus change in surface tension upon salt addition. Surface tension variation of 1-decanol in different aqueous phases upon addition of 3M NaCl was plotted against velocity (cm/sec, a.) or efficiency (b.) of the chemotaxis of a decanol droplet in these aqueous phases. The surface tension values were obtained by pendant drop tensiometry and performance by image analysis of droplet chemotaxis in Petri dish experiments. Velocity estimation was obtained from videos of droplet chemotaxis (see Methods). Efficiency was calculated by multiplying velocity for a variable obtained dividing the total path (from the starting droplet position and the salt addition point) for the droplet path (from the starting to the end position of the droplet).

Supplementary Figure S5

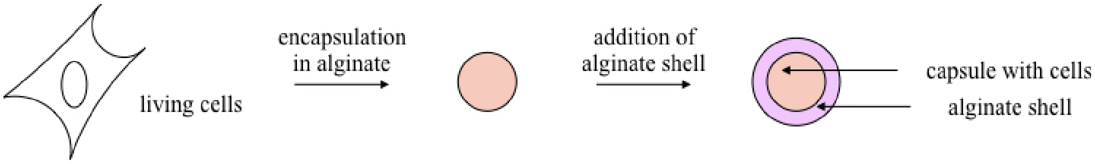

Schematic of double encapsulation . HEK293T and HS 68 cells are grown and harvested, then encapsulated in alginate to form capsules. After formation, the capsules are picked up one by one using sterile tweezers and rolled on an alginate gel and then cross-linked to create a hydrogel shell surrounding the capsule.

Supplementary Figure S6

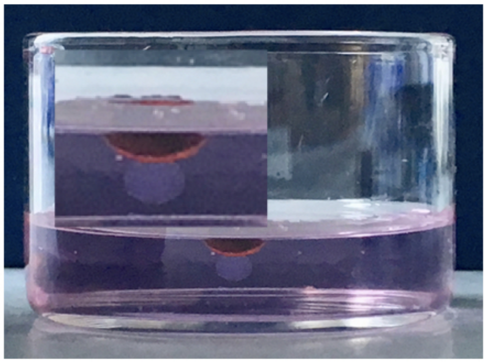

Photo of the bi-phase association test. A photo taken of the droplet-capsule association from the side showing the entire system. The droplet is colored red and sits at the air-water interface. The alginate capsule is the turbid sphere shown in its typical position at the bottom side of the droplet. Diameter of the glass dish is 4 cm.
